# Cancer classification using machine learning and HRV analysis: preliminary evidence from a pilot study

**DOI:** 10.1038/s41598-021-01779-1

**Published:** 2021-11-16

**Authors:** Marta Vigier, Benjamin Vigier, Elisabeth Andritsch, Andreas R. Schwerdtfeger

**Affiliations:** 1 Division of Oncology, Medical University of Graz, Auenbruggerplatz 15, 8036 Graz, Austria; 2 Institute of Psychology, University of Graz, Graz, Austria; 3 Independent Researcher, Graz, Austria; 4 BioTechMed Graz, Graz, Austria

**Keywords:** Cancer, Medical research, Oncology, Machine learning

## Abstract

Most cancer patients exhibit autonomic dysfunction with attenuated heart rate variability (HRV) levels compared to healthy controls. This research aimed to create and evaluate a machine learning (ML) model enabling discrimination between cancer patients and healthy controls based on 5-min-ECG recordings. We selected 12 HRV features based on previous research and compared the results between cancer patients and healthy individuals using Wilcoxon sum-rank test. Recursive Feature Elimination (RFE) identified the top five features, averaged over 5 min and employed them as input to three different ML. Next, we created an ensemble model based on a stacking method that aggregated the predictions from all three base classifiers. All HRV features were significantly different between the two groups. SDNN, RMSSD, pNN50%, HRV triangular index, and SD1 were selected by RFE and used as an input to three different ML. All three base-classifiers performed above chance level, RF being the most efficient with a testing accuracy of 83%. The ensemble model showed a classification accuracy of 86% and an AUC of 0.95. The results obtained by ML algorithms suggest HRV parameters could be a reliable input for differentiating between cancer patients and healthy controls. Results should be interpreted in light of some limitations that call for replication studies with larger sample sizes.

## Introduction

Worldwide, cancer still is the second most prevalent cause of mortality^1^. Several studies have shown that three basic biological mechanisms are involved in tumourigenesis: oxidative stress, inflammation and excessive sympathetic activity^2–4^. Oxidative stress leads to both DNA damage, the primary cause of tumourigenesis, and uncontrolled cell proliferation^5^. Inflammation enhances cancer cell resistance to stress and apoptosis. Furthermore, inflammation contributes to angiogenesis and metastasis and promotes tumourigenesis in the early stages of oncogenesis^6,7^⁠ and disease progression in its later stages^8^. Additionally, the inflammatory microenvironment, which plays a role in fighting and eliminating tumours, may also facilitate tumour growth and the production of free radicals to further induce oxidative stress. Finally, metastasis development is under the control of the sympathetic nervous system by stimulating cancer cell migratory capacity^8^.

One common factor influencing all three mechanisms is the vagus nerve as a major constituent of the parasympathetic nervous system, indexed by heart rate variability (HRV). Several studies indicate a bidirectional link between the vagus nerve and cancer. For example, it has been reported that the vagus nerve may exercise a neuromodulatory influence on cancer by slowing tumour development and progression^5,9^. Specifically, these authors inferred that vagal influences might reduce oxidative stress, modulate inflammation, and inhibit sympathetic activity. Notably, the information about tumourigenic activity related to tumour-associated proinflammatory cytokines is transferred to the brain by the vagus nerve^10,11^. Studies involving patients with vagotomy confirmed the role of the vagus nerve in cancer onset, showing an increased risk of developing lung or colorectal cancer after the surgery^12,13^. On the other hand, Strous et al.^14^⁠ suggested that the same cancer-related mechanisms accompanying the development and progression of a malignant tumour may cause vagal dysfunction and decreased HRV. Although the origin of the relationship between the vagus nerve and cancer is unclear, the lower HRV was unanimously reported in early and advanced cancer patients compared to healthy individuals^15,16^.

Vagus nerve activity can be quantified by recording an electrocardiogram (ECG) and analysing beat-to-beat fluctuations in heart rate. HRV parameters can be described by linear (time- and frequency-domain) and non-linear measures^17^.

Time-domain measures of HRV quantify the amount of variance in the RR-intervals, which represents the period between successive heartbeats. Time-domain statistics are analysed through parameters such as the standard deviation of RR-intervals in a defined time period, the root mean square of successive differences (RMSSD) indicating short-term fluctuations in successive RR-intervals, the number or proportion of different pairs of successive RR-intervals that differ by more than a fixed time interval^18^.

Frequency-domain measures quantify the distribution of absolute or relative power into four frequency bands established by the Task Force of the European Society of Cardiology and the North American Society of Pacing and Electrophysiology (1996): ultra-low-frequency (ULF ≤ 0.003 Hz), very-low-frequency (VLF: 0.0033–0.04 Hz), low-frequency (LF: 0.04–0.15 Hz), and high-frequency (HF: 0.15–0.4)^19^. Frequency-domain measures are analysed by power spectral density computation using several parametric or nonparametric methods in different frequency bands of interest^20^.

Finally, non-linear measures of HRV quantify the unpredictability of fluctuations in a time series, as the HRV signals are non-linear and non-stationary by nature^18^. Moreover, HRV parameters, when examined using chaos theory and non-linear system theory, suggest the non-linear mode of interaction between the mechanisms involved in cardiovascular regulation^18^.

The differences between healthy individuals and cancer patients in time and frequency domain measures of HRV have often been reported, ultimately showing a vagal impairment in the latter group^16,21^. A lower HRV in cancer patients indicates autonomic dysfunction, which most cases exhibit^16,21,22^. This cancer-related alteration is characterised by a sympathovagal imbalance with highly active SNS and impaired PNS functioning^23^. Although not often examined in cancer, the changes in non-linear HRV measures were reported as early signs of several diseases^24^. Therefore, we explored the combination of several linear and non-linear HRV features to classify cancer vs non-cancer.

Machine Learning (ML) established an essential role in healthcare and medical research^25,26^. In cardiovascular research, ML has been successfully used in automated ECG analysis for arrhythmia detection and classification, ischemia detection, left and right ventricular hypertrophy, bilateral ventricular hypertrophy, and diabetes^27–30^. Several studies classified disorder-affected individuals vs healthy controls based on HRV and machine learning algorithms. For example, Aggarwal et al.^31^ classified healthy vs diabetic rats based on diabetes-related changes in HRV, using an artificial neural network (ANN) and support vector machine (SVM). Other researchers created a neural network that automatically classified diabetic and healthy individuals based on disease-related HRV alterations^32^.

To the best of our knowledge, only two studies applied machine learning to predict or classify cancer based on HRV analysis. Shukla and Aggrawal^33^ extracted HRV indices from ECG recordings of 104 lung cancer patients and 30 healthy individuals. The authors found reduced HRV in cancer patients compared to healthy people. Further, the authors reported that the decrease in HRV levels was related to the severity of the disease. They predicted and classified lung cancer stages using ANN and SVM with 93.09% and 100% accuracy. In a recent study, Shukla, and Aggrawal^34^ analysed the 5-min electrocardiogram of 114 breast cancer patients and 13 age-matched healthy individuals. The authors used a Lavenberg–Marquardt algorithm-based artificial neural network and a support vector machine that classified two groups based on spectral HRV features with a maximum accuracy of 54.2% and 100%, respectively.

This research proposes a new methodology in cancer classification based on HRV and ML, which is different from the methods discussed above. To classify cancer vs healthy individuals, we used an ensemble model with the stacking method. Specifically, we aimed to examine the capacity of three different ML algorithms to recognise patterns in HRV to classify ECGs. Next, we aggregated the predictions from these algorithms to build a meta-classifier to improve the robustness and classification accuracy. In addition, we applied the Recursive Feature Elimination method to select the most relevant HRV features. We also addressed the issue of class imbalance.

We recruited patients with tumours most consistently related to vagal dysfunction (i.e., breast cancer, prostate cancer, colorectal cancer, lung cancer, and pancreatic cancer) and within different stages of cancer development. Our sample contains five of the six most common types of cancer in Austria^35^. Thus, patients suffering from these types of cancer are often present in general oncology units, similar to where we collected our data. Importantly, research reports reduced HRV and a decreased survival rate in different types and stages of disease^16,36,37^. Also, no clear agreement exists among researchers regarding the direction of influence between HRV and cancer and the timeline of observed cancer-related changes in HRV. Therefore, we included patients from various cancer stages to account for the possible differences in patient’s autonomic activity.

HRV measures were recorded from cancer patients and healthy individuals. A subset of time, frequency domain and non-linear features averaged over 5 min was used as input to the machine learning models created to distinguish cancer patients from healthy controls based on HRV analysis.

In particular, we investigated the performance of three ML classifiers: Random Forest (RF), Linear Discriminant Analysis (LDA), and Naive Bayes (NB). Next, we created one ensemble model based on the combination of those models using the stacking method and eXtreme Gradient Boosting (XGB) from the Caret package^38^ (Version 6.0.88) in R^39^ (Version 4.1.0).

Our study makes a new contribution to the existing research. Its foremost advantage is to employ an ensemble stacking algorithm that allows to reduce the uncertainties of predictions and to improve the robustness of classification.

## Methods

### Participants

The data set comprised two cohorts, 77 cancer patients (see Table 1) and 57 healthy controls. ECG, age, gender, and medical variables were recorded. The cancer group was age and sex-matched with the control group. Exclusion criteria included diabetes, cardiovascular pathologies, pregnancy, and psychiatric disorders. Both cancer patients and healthy individuals were excluded from the study if they were suffering from or taking any medication related to psychiatric or cardiac conditions, diabetes, and pregnancy. The average age of participants was 50 years old, and around 60% of the participants were women. Most cancer patients (42.86%) were diagnosed with breast cancer, 2.6% with prostate cancer, 3.9% with lung cancer, 37.67% with colorectal cancer, and 12.97% with pancreatic cancer.

**Table 1:** Cancer patients characteristics.

Patients characteristics	N 77
**Age** (in years)	50
**Gender**	
Male	30
Female	47
**Cancer type**	
Breast	33
Colorectal	29
Lung	3
Pancreas	10
Prostate	2
**Cancer stage**	
I	32
II	7
III	12
IV	26

Thirty-two patients were diagnosed with the first cancer stage, seven with the second, twelve with the third, and twenty-six with the fourth stage.

When we recorded the ECGs, none of the patients were receiving any cancer treatment. Moreover, we asked participants to avoid caffeine, nicotine and alcohol consumption and refrain from physical exercise in the two hours preceding the ECG recording.

The ethics committee of the Medical University of Graz approved all the study protocols. The participants gave written informed consent before taking part in the study. The procedures used in this study adhere to the tenets of the Declaration of Helsinki.

### HRV analysis and feature selection

An ECG was recorded in a sitting position for 5 min, applying a chest lead.

In cancer patients, an ECG Holter monitoring was applied (Schiller Holter MedilogAR). Three Ag/AgCl electrodes were placed on the distal end of the right clavicle, lower left rib cage chest, and lower abdomen.

Control group recordings were obtained using eMotion Faros 180°, Mega Electronics Ltd. Both devices are medically certified, highly accurate, and widely used in research.

For both groups, the signal was sampled at a rate of 1,000 Hz. HRV was analysed offline via Kubios HRV Premium software (version 3.3.1)^40^ following International Guidelines^19^. The software is device-independent, ensuring similar treatment of ECG signal and artifacts^41^. “Before R-wave time instant extraction, the R-wave is interpolated at 2000 Hz to improve the time resolution of the detection”^41^.

Visual artefact correction was performed on the raw IBI series, and if needed, an automatic correction algorithm was applied. 6.7% of the recordings were affected by artefacts. According to Kubios guidelines^40^, we included no recording containing 5% or more artefacts in the analysis. Time-domain HRV features were calculated directly from the time series of RRIs. Frequency-domain analysis was fast Fourier transform (FFT) based, and the non-linear parameters were computed from the detrended RR interval data.

We decided to select twelve HRV features for two reasons. First, several studies reported impaired autonomic activity and significantly decreased vagal functioning based on some time and frequency HRV features in cancer patients^36,37,42^. As a consequence, it seemed important to focus on the HRV features most consistently associated with cancer-related alternations. Second, disease-related changes in non-linear HRV indices were used to detect some disorders at their early stages^43–47^. Hence, they might improve cancer classification in the early stages. Based on prior knowledge and clinical plausibility, we selected twelve HRV features as described in Table 2. We compared the healthy sample with cancer patients by applying the Wilcoxon sum-rank test.

**Table 2:** HRV features.

HRV features	Type	Description
Mean RR (ms)	Time-domain	The average of RR intervals during a period of time
SDNN (ms)	Time-domain	Standard deviation of NN intervals
RMSSD (ms)	Time-domain	Root mean square of successive RR interval differences
pNN50%	Time-domain	Percentage of successive RR intervals that differ by more than 50 ms
HRV triangular index	Time-domain	The integral of the sample density distribution of RR intervals divided by the maximum of the density distribution
TINN (ms)	Time-domain	Baseline width of the RR interval histogram
LF power %	Frequency-domain	Includes the frequency range between 0.04 Hz and 0.15 Hz
HF power %	Frequency-domain	Includes the frequency range between 0.16 Hz and 0.4 Hz
Total Power (ms)	Frequency-domain	Reflects the overall autonomic activity
SD1	Non-linear	Poincaré plot standard deviation perpendicular to the line of identity
SD2	Non-linear	Poincaré plot standard deviation along the line of identity
Sample Entropy	Non-linear	Measures the regularity and complexity of a time series

Next, we used the Recursive Feature Elimination (RFE) method to obtain optimal performance for the classifiers and chose the best combination of features from our prior knowledge-based choice. RFE is a wrapper-type feature selection algorithm, that applies a backward selection process to find the most advantageous sequence of features. The first step for RFE is to construct a model based on all features and estimate the importance of each feature in the model. Then, it rank-orders the features and eliminates those with the lowest importance iteratively based on model evaluation metrics^48^. In this research, we used the random forest algorithm wrapped by RFE and applied it to select HRV features.

### Model development

Model development was carried out with five HRV features selected by RFE and the outcome was a dichotomous variable with two levels: patient and control. We standardised the data using the following options from Caret’s pre-process function: BoxCox, center and scale. The BoxCox method allows for correcting the skewness of the data. Centering involves subtracting mean from values, while scaling enables to divide values by standard deviation.

Our model development consisted of two steps. First, we chose three base models that incorporated various underlying algorithms. Second, we created an ensemble based on the stacking method to improve the accuracy and robustness of classification. Stacking is a well-established and powerful ensemble machine learning technique. In the stacked ensemble model, a meta-classifier is trained using the predictions of the base classifiers to make an optimal combination of the predictions. The stacking method integrates base learners' strengths to attain more robust performance, reduce estimation uncertainties and improve prediction accuracy^49^.

In the present research, HRV features were supplied into the base models, producing the predictions used by the meta-classifier, which classified the input data into “cancer “or “patient” categories.

We applied the three following machine learning algorithms to classify cancer and healthy individuals: Linear Discriminant Analysis (LDA), Naïve Bayes (Nb), and Random Forest (RF). All the ML methods are included in the Caret R package.

LDA is a classification method that searches for a linear combination of variables that best divides two classes. Nb classifier is a probabilistic machine learning model based on the Bayes theorem used for the classification task. RF is a machine learning classification algorithm that builds a decision tree model^48^.

We implemented tenfold cross-validation with five repeats to assess three diverse classification models available in the Caret package using 60% of the dataset for training. This method randomly splits the dataset into ten segments where one segment represents the validation set, and the remaining nine parts are employed to build the training set. This operation is repeated ten times. Each time one part is removed, and thus we obtain a different part of the data for validation. The average result of the ten parts consists of the final prediction result^48^. We used 40% of the entire dataset as an independent testing set for the final model evaluation.

The training set contained 35 control and 47 cancer individuals. To deal with the class imbalance, we employed an up-sampling technique offered by the Caret package. This method randomly replicates the instances in the minority class by sampling with replacement to have the same size.

We used the default tuning hyper-parameter optimisation approach in the R package Caret, a grid search assessing three different sets of values for the hyper-parameters and selecting the best performing values for the final model.

To evaluate if any improvement in the performance of base-classifiers was possible, we created an ensemble model based on the stacking method. The stacked meta-model comprised the predictions of all three base classifiers. We applied Extreme Gradient Boosting as an ensemble algorithm to create the stacked model.

We used the following R packages to build the stacked ensemble model: Caret (Version 6.0.8)^38^ and caretEnsemble^50^ (Version 2.0.1).

We evaluated the models’ performance using metrics including accuracy, sensitivity (true-positive rate), specificity (true negative rate), Cohen’s Kappa coefficient and ROC. A confusion matrix was created for the stacked ensemble model.

### Ethics approval

Approval was obtained from the ethics committee of Medical University of Graz. The procedures used in this study adhere to the tenets of the Declaration of Helsinki.

## Results

### HRV analysis

HRV variables were compared between cancer and healthy individuals using Wilcoxon sum-rank test. We observed significantly lower values (and for most of the measures, considerably lower *SD*s) in cancer patients as compared to healthy controls. The results for both groups are summarised in Table 3.

**Table 3:** HRV features comparison between cancer and control individuals.

HRV features	Cancer (*M*/*SD*)	Control (*M*/*SD*)	*W*	*p*
Mean RR (ms)	717.91/93.18	832.9/108.9	5119	< 0.001
SDNN (ms)	21.4/7.57	35.37/14.76	5289	< 0.001
RMSSD (ms)	13.87/4.49	29.26/16.47	5332	< 0.001
pNN50%	0.62/0.87	7.82/12.15	5345	< 0.001
HRV triangular index	5.5/1.73	8.95/3.57	5503.5	< 0.001
TINN (ms)	126.69/57.68	194.6/101.61	4625	0.013
LF power %	69.27/12.22	60.91/17.85	5160	< 0.001
HF power %	16.69/9.17	28.92/18.2	5522	< 0.001
Total power (ms)	440.23/357.83	1294/1231.43	5425	< 0.001
SD1	9.81/3.18	20.72/11.66	5332	< 0.001
SD2	28/10.55	45.03/18.6	5290	< 0.001
Sample entropy	1.37/0.34	1.58/0.32	4811	0.002

### Feature selection

We ran the recursive feature elimination algorithm using 12 prior knowledge-based selected features and individuals' health status (healthy/cancer) as an outcome variable. The five most important features were selected and all other measures were eliminated.

**Figure 1:**
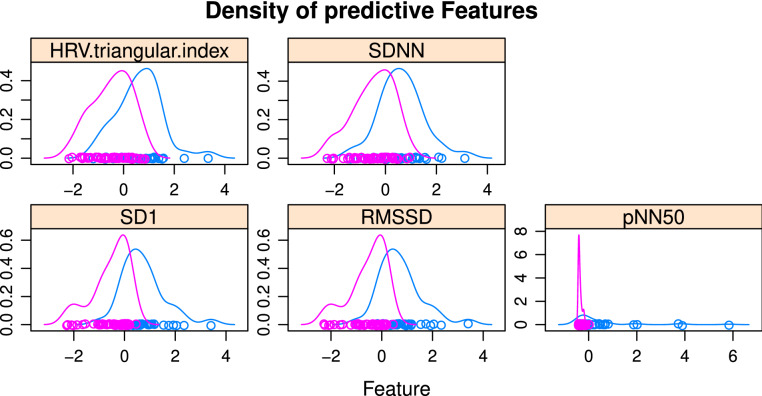
Density plots for the five most important HRV features selected by the RFE method. The density plots illustrate the distribution of the top features between control individuals (pink) and cancer patients (blue).

Table 4 shows the results of RFE with the five most important features. Figure 1 represents density plots for the five top HRV features selected by RFE. None of these features shows a significant overlap suggesting that all might constitute relevant input for classification.

**Table 4:** Results of recursive features elimination algorithm applied to 12 prior knowledge-based selected HRV features.

Variables	Accuracy	Kappa	Accuracy SD	Kappa SD	Selected
4	0.8	0.58	0.1	0.21	
8	0.83	0.64	0.1	0.2	*
12	0.82	0.62	0.1	0.21	
Top 5 out of 8	RMSSD, SD1, SDNN, pNN50, HRV.triangular.index

### Model performance

#### Performance of base-classifiers

**Figure 2:**
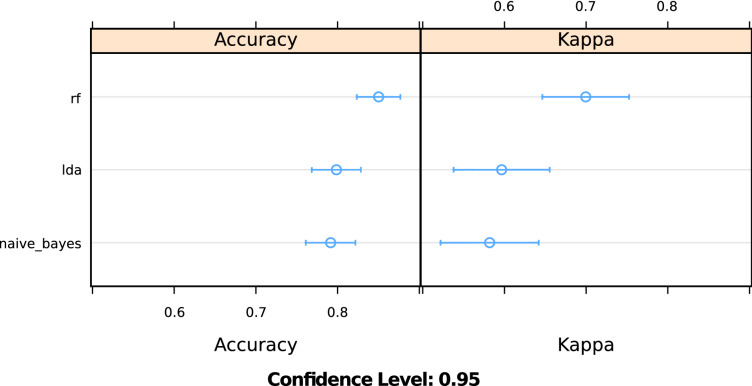
Accuracy and Kappa statistics for different classifiers.

**Figure 3:**
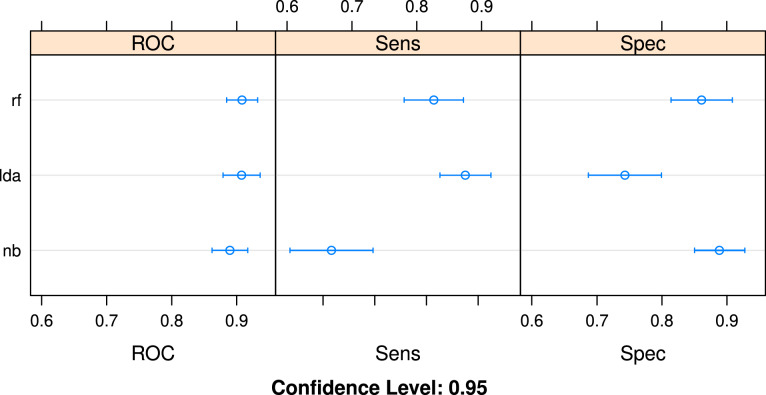
ROC, sensitivity and specificity for different classifiers.

The results shown in Table 5, Figs. 2 and 3 indicate the performance metrics of three base classifiers on the training set with ten-fold cross-validation. They suggest that the algorithm of Naive Bayes performed the worst on the classification task, showing an accuracy of 79%. The algorithm of Linear Discriminant Analysis performed slightly better, with an accuracy of 80%. The algorithm of random forest performed best, with 85% of accuracy.

**Table 5:** Prediction performance of base classifiers and stacked ensemble.

Classifier	Accuracy	Kappa	ROC	Sensitivity	Specificity
LDA	0.798	0.6	0.91	0.88	0.74
NB	0.790	0.58	0.89	0.67	0.89
RF	0.849	0.7	0.91	0.83	0.86
Ensemble	0.93	0.86	0.96	0.85	0.92

**Figure 4:**
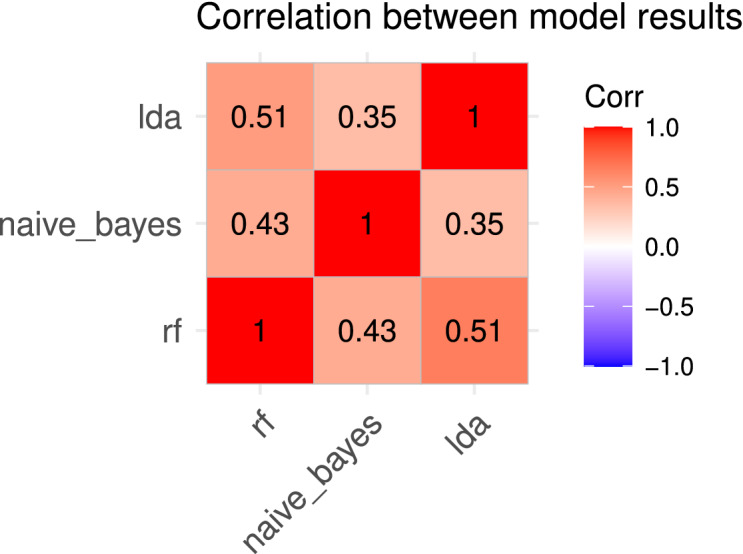
Correlation between the results of base classifiers.

The correlation between predictions of models was moderate, ranging from 0.35 to 0.51 (see Fig. 4). Thus suggesting that every classifier might perform better at learning different data patterns. In addition, all three base classifiers displayed promising performances while classifying cancer vs healthy individuals based on HRV analysis. Thus, we integrated all models to build a meta-classifier and analyse if the classification accuracy improved.

### Performance of meta-classifier

**Figure 5:**
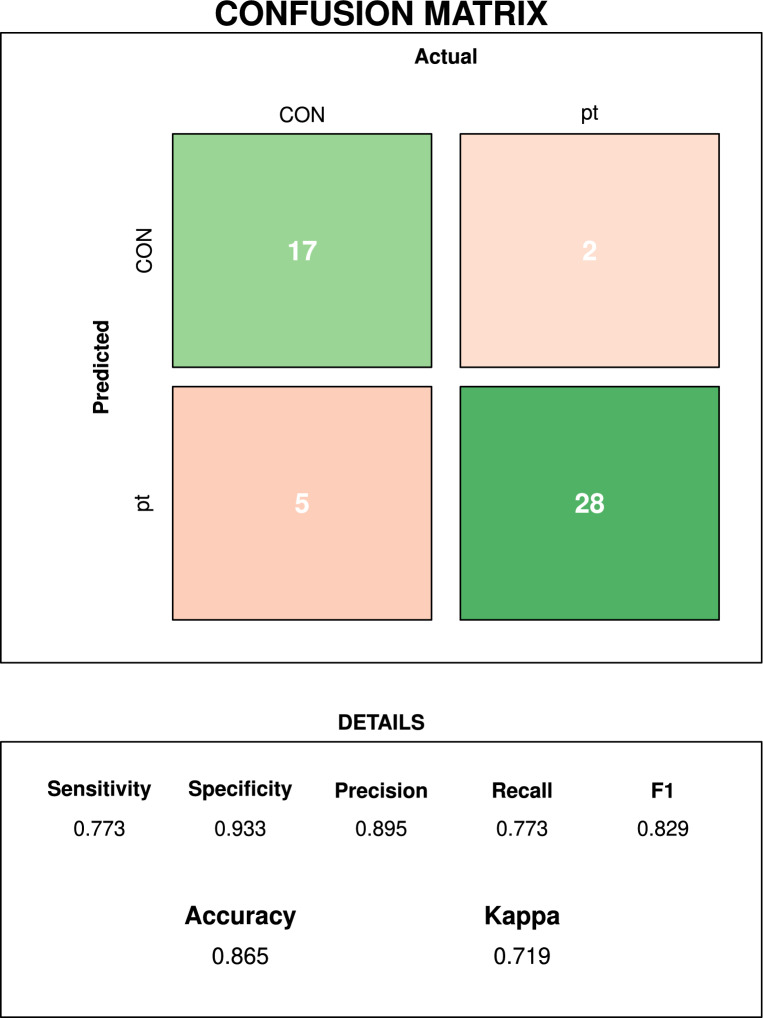
Confusion matrix showing the meta-classifier results for the testing dataset.

In a final step, after training and evaluating models created using the individual ML algorithms, we devised a stacked model aggregating the predictions of the base classifiers. This meta-model was trained using Extreme Gradient Boosting. The stacked model produced an accuracy of 0.929 and Kappa of 0.859 with ROC = 0.956, sensitivity of 0.846, and specificity of 0.924, thus outperforming all base learners (see Table 5). On the test set (unseen data), the model had an accuracy of 0.865 and a Kappa of 0.719 (see Fig. 5), sensitivity of 0.773 and specificity of 0.933.

The confusion matrix for the meta-classifier presents the performance of this model on the testing set (Fig. 5). The outcome variable has two values (patient and control). The column with control values shows that 17 control data points were correctly classified by the model, whereas two were incorrectly classified as belonging to the opposite class (patients). In the case of patients, the model correctly recognised 28 cases and wrongly assigned five patients to the control class.

**Figure 6:**
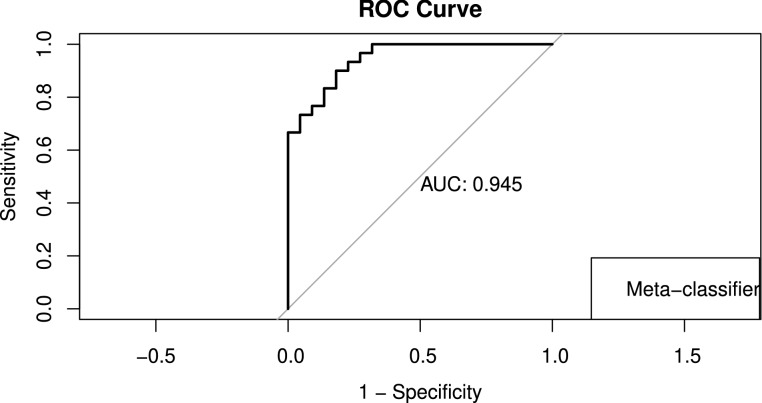
Receiver operating characteristic curve for the meta-classifier model on the testing dataset. The horizontal axis represents the false-positive rate (1-Specificity). The vertical axis represents the true-positive rate (Sensitivity).

The ensemble model achieved an area under the receiver operating characteristics (ROC) curve (AUC) of 0.945 (95% CI 0.8916–0.9993), see Fig. 6.

## Discussion

This pilot study evaluated the possibility of machine learning-based discrimination between cancer patients and healthy controls based on five-minute-ECG recordings. In the first step, comparing HRV parameters (linear and non-linear features) between cancer patients and healthy controls suggested significant differences in all HRV parameters constituting an input for the machine learning in this research. These findings align with several studies^16,21^ documenting decreased levels of HRV in cancer patients compared to healthy populations. Specifically, a systematic review^22^ including 19 studies conducted in 2018 concludes that decreased HRV illustrates a disease-related autonomic dysfunction in cancer patients.

Importantly, the performance of three well-established machine learning algorithms achieved satisfactory results ranging from 79 to 85% accuracy. The RF performed best compared to the other ML algorithms in this study. The differences in accuracy between the base classifiers were, however, modest. There was a slight variation in accuracy between the best (RF) and worst (NB) performing algorithms (0.059).

To improve the robustness and accuracy of the classification model, we created a stacked learner that included the predictions of all three individual ML models. The accuracy of the stacked model was 8% higher compared to the RF model. Therefore, we conclude that in this study, a slight improvement in accuracy was achieved by stacking multiple classification models.

On the testing set, the meta-learner's performance was still satisfactory. However, the model’s sensitivity was pretty moderate (77%). Thus, one should exert caution while excluding cancer based on this classification model. At the same time, the meta-classifier performed very well in terms of specificity (93%). It could be, therefore, speculated that our model might be accurate while used to confirm a suspected cancer diagnosis. The model’s high specificity might imply low chances of getting positive results in non-cancer individuals. Considering this model’s moderate sensitivity, it might be used as a complementary tool in oncology and applied when, based on other tests, there is a suspicion of cancer.

Both sensitivity and specificity of the meta-classifier are greater than 70%, which seems to be a reasonable trade-off^51^. Nevertheless, at this stage of the research, it is uncertain if the results might be generalised for different algorithms beyond the ML used in this analysis. Our meta-classifier did not notably outperform the results from the best base classifier. Notwithstanding, using an ensemble model based on different ML algorithms may prevent reduced performance and prediction uncertainty.

Despite the moderate improvement in classification accuracy of the ensemble model, it should be noted that the performance of our model proved satisfactory as compared with previous research, showing 86% accuracy, 93% specificity and 77% sensitivity while classifying cancer vs healthy individuals on unseen data.

Only two studies employed machine learning and HRV analysis to classify healthy individuals vs cancer patients. Shukla and Aggrawal^33^ predicted and classified lung cancer stages using ANN and SVM with 93.09% and 100% accuracy. The same authors^34^ applied a Lavenberg–Marquardt algorithm-based artificial neural network (ANN) and support vector machine (SVM) to classify cancer vs healthy individuals based on spectral features of HRV, with maximum accuracy of 54.2% and 100%, respectively.

Some differences between these studies and the present work are worth mentioning. First, the studies are different in terms of statistical analysis. We created a stacked ensemble model while the authors of the research mentioned above applied single ML algorithms.

Moreover, our study did not focus on classifying between different cancer stages. Further, we decided to recruit patients with different cancer types most consistently related to vagal dysfunction (i.e., breast cancer, prostate cancer, colorectal cancer, lung cancer, and pancreatic cancer).

In contrast, previous research selected one type of cancer. Although similar in sample size with our research, both studies applied ML on highly imbalanced data without accounting for it. Finally, we applied the Recursive Feature Elimination method of feature selection.

Therefore, the findings of this pilot study could constitute a preliminary framework for developing cancer classifications techniques based on HRV analysis and ML. It should be noted, though, that further research is necessary to evaluate the algorithm’s sensitivity for different cancer stages. In this study, most of the patients were ascribed to stage 1 and stage 4, respectively. Thus, more research, including patients with different cancer stages, is needed.

While the findings of this early pilot study illustrate a satisfactory performance of machine learning algorithms to classify cancer vs healthy individuals from HRV measures, several limitations should be noted.

First, the sample size was relatively small due to clinical routines and the time needed for acquiring HRV data, and the recording time was not standardised. The ECG was recorded at the same location for each patient cohort; however, the measurements took place at a different time of the day for all the participants. Hence, circadian rhythms could have increased variance in the measures. Future research should aim to standardise the time of recordings to control for circadian rhythms. Also, sleep alterations can cause a change in HRV features^52^. Thus, future research should evaluate the cancer patients' sleep patterns via standard questionnaires.

Second, we used two different devices to record cancer patients’ and healthy individuals’ ECG, which might have introduced some differences. However, in both devices, the signal was sampled at the same rate, avoiding differences in the accuracy of HRV estimation. Both devices are medically certified, highly accurate, and widely used in research. Moreover, we based our analysis on HRV values calculated in and exported from Kubios software, assuring similar treatment of ECG signal and artifacts^41^.

Third, in this study, we focused on certain cancers most consistently associated with HRV dysfunction^2,42,53,54^. Future studies should aim for larger and more diverse samples of cancer patients and healthy controls, which might help to probe the robustness of cancer detection based on HRV analysis.

Fourth, it should be highlighted that although cancer patients and healthy controls were age and gender-matched, other potential confounds, like physical activity, smoking, body weight, etc.^55^ could have contributed to the differences between groups. As far as such differences are representative for the group of cancer patients and reflect common risk factors for cancer (e.g., a higher number of smokers, lower levels of physical activity, obesity)^56^, the classification based on ML remains valid.

Fifth, despite a diverse selection of ML algorithms, there is a possibility that better results could have been achieved with other ML algorithms.

Sixth, although we used the default hyper-parameters provided by the Caret package, tuning might improve classification performance.

Additionally, the choice of the algorithm applied in the stacking ensemble might impact the results. Future work should focus on exploring different stacking algorithms (i.e., generalised linear model). Finally, although a comparison of different diseases (e.g., diabetes, cardiovascular pathologies, mental disorders) associated with aberrations in HRV was beyond the scope of this research (see, e.g.^57^ for a review of HRV in severe clinical conditions reflecting brain–heart interaction), future research should aim to identify distinct patterns of HRV for each of these conditions and attempt a multi-class classification based on HRV analysis and machine learning techniques.

## Conclusion

In this pilot study, we demonstrated preliminary results illustrating machine learning-based cancer classification vs healthy individuals, using linear and non-linear HRV measures. In line with the previous research on HRV in cancer patients^2,4,16,58^ the findings confirm significant differences in autonomic function compared to healthy controls. Furthermore, we demonstrated that ML algorithms could classify healthy vs cancer individuals based on ECG with acceptable accuracy, sensitivity, and specificity compared to previous studies. We also found that we could create a meta-classifier that performed slightly better than underlying base-classifiers by using a stacking method. Random Forest led to the highest predictive accuracy, and Naïve Bayes performed worst compared to the other algorithms. The stacked model accuracy was about 8% higher than the best individual model. Ensemble model performance on unseen data was satisfactory (accuracy of 86%), showing a moderate sensitivity (77%) and high specificity (93%). Notably, the moderate sensitivity of this model suggests that ruling out cancer based on this classification method should be done with caution. At the same time, the meta-classifier performed very well in terms of specificity, suggesting high performance when aiming to confirm a suspected cancer diagnosis. Future studies may shed light on how ML and HRV analysis could be of practical value as a supplementary tool in oncology.

## Abbreviations

AUC: Area under the curve
ECG: Electrocardiogram
FFT: Fast Fourier transform
RFE: Recursive features elimination
RF: Random forest
HRV: Heart rate variability
IBI: Interbeat interval
ML: Machine learning
XGB: EXtreme gradient boosting
NB: Naïve Bayes
LDA: Linear discrimination analysis
